# Treatment of adult MPSI mouse brains with IDUA-expressing mesenchymal stem cells decreases GAG deposition and improves exploratory behavior

**DOI:** 10.1186/1479-0556-10-2

**Published:** 2012-04-20

**Authors:** Flávia Helena da Silva, Vanessa Gonçalves Pereira, Eduardo G Yasumura, Lígia Zacchi Tenório, Leonardo Pinto de Carvalho, Bianca Cristina Garcia Lisboa, Priscila Keiko Matsumoto, Roberta Sessa Stilhano, Vivian Y Samoto, Bruno Frederico Aguilar Calegare, Letícia de Campos Brandão, Vânia D’Almeida, Thaís RM Filippo, Marimélia Porcionatto, Leny Toma, Helena Bonciani Nader, Valderez Bastos Valero, Melissa Camassola, Nance Beyer Nardi, Sang Won Han

**Affiliations:** 1 Department of Genetics, Universidade Federal de Rio Grande do Sul, Porto Alegre, Brazil; 2 Research center for gene therapy, Universidade Federal de São Paulo, São Paulo, Brazil; 3 Department of Pediatrics, Universidade Federal de São Paulo, São Paulo, Brazil; 4 Department of Psychobiology, Universidade Federal de São Paulo, São Paulo, Brazil; 5 Department of Biosciences, Universidade Federal de São Paulo, São Paulo, Brazil; 6 Department of Biochemistry, Universidade Federal de São Paulo, São Paulo, Brazil; 7 Laboratory of stem cells and tissue engineering, Universidade Luterana do Brasil, Porto Alegre, Brazil; 8 Department of Biophysics, Universidade Federal de São Paulo, Rua Mirassol 207, São Paulo, 04044-010, Brazil

**Keywords:** MPSI, Gene therapy, Retroviral vectors, Lysosomal storage disorder, Mesenchymal stem cell, IDUA

## Abstract

**Background:**

Mucopolysaccharidosis type I (MPSI) is caused by a deficiency in alpha-L iduronidase (IDUA), which leads to lysosomal accumulation of the glycosaminoglycans (GAGs) dermatan and heparan sulfate. While the currently available therapies have good systemic effects, they only minimally affect the neurodegenerative process. Based on the neuroprotective and tissue regenerative properties of mesenchymal stem cells (MSCs), we hypothesized that the administration of MSCs transduced with a murine leukemia virus (MLV) vector expressing IDUA to IDUA KO mouse brains could reduce GAG deposition in the brain and, as a result, improve neurofunctionality, as measured by exploratory activity.

**Methods:**

MSCs infected with an MLV vector encoding IDUA were injected into the left ventricle of the brain of 12- or 25-month-old IDUA KO mice. The behavior of the treated mice in the elevated plus maze and open field tests was observed for 1 to 2 months. Following these observations, the brains were removed for biochemical and histological analyses.

**Results:**

After 1 or 2 months of observation, the presence of the transgene in the brain tissue of almost all of the treated mice was confirmed using PCR, and a significant reduction in GAG deposition was observed. This reduction was directly reflected in an improvement in exploratory activity in the open field and the elevated plus maze tests. Despite these behavioral improvements and the reduction in GAG deposition, IDUA activity was undetectable in these samples. Overall, these results indicate that while the initial level of IDUA was not sustainable for a month, it was enough to reduce and maintain low GAG deposition and improve the exploratory activity for months.

**Conclusions:**

These data show that gene therapy, via the direct injection of IDUA-expressing MSCs into the brain, is an effective way to treat neurodegeneration in MPSI mice.

## Introduction

The disruption of lysosomal activity during cellular metabolism results in an accumulation of glycosaminoglycans (GAGs), which leads to a group of inherited diseases known as lysosomal storage disorders (LSDs). Mucopolysaccharidosis I (MPSI) is a LSD in which two GAGs, dermatan and heparan sulfate, accumulate due to a deficiency in alpha-L iduronidase (IDUA - EC 3.2.1.76). The frequency of MPSI is 1 in 100,000 live births, and it presents as a syndrome with one of three phenotypes, Hurler (OMIM #607014), Hurler-Scheie (OMIM #607015) or Scheie (OMIM # 607016). Recently, it has been suggested that patients with MPSI should be classified into two case types, attenuated (Hurler-Scheie and Scheie) and severe (Hurler) [1]. MPSI patients develop splenomegaly, bone and articular diseases and cardiorespiratory malfunctions, among other problems. Hurler syndrome also results in progressive and irreversible neurodegeneration, which leads to death in early childhood [2].

Two types of treatment are currently available for MPSI. The first option is bone marrow or stem cell transplantation, which is efficient but limited by the scarcity of compatible donors, the high mortality rate of the procedure itself and the high financial cost. The second alternative is enzyme replacement therapy (ERT), which is effective in decreasing the hepatosplenomegaly and improving articular movement. However, neither transplantation nor ERT have prevented neurodegeneration [3]. Although preclinical studies have shown that the enzyme can reach the central nervous system (CNS) and provide therapeutic benefits [4] following a high-dose infusion, these levels are not attainable at the clinical level because more frequent infusions and higher amounts of enzyme would be necessary. Therefore, even the initiation of ERT early in childhood will not result in significant benefits to the CNS, despite an improvement in the visceral disease. In addition, MPSI syndrome is not routinely diagnosed in newborns, and most of the affected children are diagnosed much later in life [3]. These observations necessitate the development of alternative procedures aimed specifically at treating the CNS.

Intravenous gene transfer into the brain of animals with LSD via viral vectors is feasible in neonates, as has been demonstrated with adenovirus-associated viral (AAV) vectors, lentiviral vectors and retroviral vectors [5-8]. In adult MPSI mice, however, the systemic injection of AAV and MLV (murine leukemia viral vector) has been shown to result in a significant serum enzyme level, but very low enzymatic activity has been detected in the brain [6,8-11]. Importantly, a significant improvement in the neurological abnormalities, as demonstrated using behavioral tests, was only observed after neonatal viral gene transfer in a murine MPSI model [5]. However, the best way to reduce GAG accumulation in the brain appears to be through the administration of AAV vectors locally, even though this procedure is riskier and more complicated in clinical trials [9,12].

To date, no gene therapy studies have examined the efficacy of treating adult mice at different stages of disease, which is a more appropriate model if we want to translate the therapies into clinical trials. In few month-old MPSI mice, a progressive decline in behavior is observed [12] due to an accumulation of GAG deposits in the brain. How GAG deposits affect neurocognition remains unclear, even in patients [1]. Thus, it will be of great importance to include behavioral tests that accurately reflect the changes in GAG deposition, especially in brain gene therapy studies for MPSI. Behavioral tests have already been performed in other types of MPS and show a good correlation with changes in GAG deposition [7,13-16].

Mesenchymal stem cells (MSCs) have the potential to differentiate into various cell types of mesenchymal origin, such as osteoblasts, chondrocytes and adipocytes [17,18]. Additionally, recent work has demonstrated that MSC can differentiate into neural cells *in vitro* and *in vivo*[19,20]. A therapeutic effect from MSCs in an injured brain has been observed in several animal models and clinical trials [21,22]. The benefits of MSC therapy seems to stem from the role of MSCs in neuroprotection, the promotion of tissue regeneration, the induction of growth factor and cytokine expression and vascularization. MSC-based therapy has also been used in mice with MPS VII [23].

Based on the characteristics of MSCs and the need to provide additional neurotrophic factors to mice with MPSI *in situ*, we hypothesized that MSCs modified to express IDUA delivered directly into the brain ventricles of mice with MPSI would produce a synergistic therapeutic effect, especially in adult animals. To validate our hypothesis, adult mice with MPSI were used in our gene therapy study for evaluating locomotor and exploratory behavior functions in addition to molecular analyses.

## Materials and methods

### *IDUA* −/− mice

*IDUA* −/− (KO) mice were generated via the targeted disruption of the *IDUA* gene [23] (kindly provided by Dr. Elizabeth Neufeld, UCLA, Los Angeles, CA, USA) and maintained by breeding heterozygous animals. The Research Ethics Committee of the Federal University of São Paulo approved all of the conditions and experimental protocols (approval number CEP 1201/07). In our experiments, 12-week-old (12 w) and 25-week-old (25 w) KO mice were used, and these mice were euthanized at 20 weeks (20 w) and 29 weeks (29 w), respectively, for biochemical and histological analyses.

### MLV vectors, mesenchymal stem cell cultures and transduction

The cDNAs encoding either human IDUA or GFP were excised from pTIGER vectors (http://www.addgene.org) using EcoRI and inserted into an EcoRI-digested pLXSN vector (24). The resulting plasmids were designated MLV-IDUA and MLV-GFP. The pLXSN vector has a retroviral long-terminal repeat sequence (L), cloning site (X), internal Simian virus 40 promoter (S) and neomycin-resistance gene (N).

To obtain virus-producing cells, 5×10^5^ PT67 packaging cells (Invitrogen) were transfected using calcium phosphate. After 6 hours, the medium was replaced with fresh medium, and the cells were cultured for an additional 48 h in a humidified incubator at 37°C. The cultured cells were split for G418 selection (1 mg/ml), and the virus-producing clones were isolated using cloning rings for amplification. The vectors were titrated using NIH3T3 cells and 8 μg/mL of polybrene [24,25].

The viral vectors were collected from the supernatant of each cell line and concentrated in a Sorvall centrifuge (rotor SS34) at 16000 rpm for 2 hours [24,25]. For each 20 mL of viral vector, 4 mL of 20% sucrose in water was added. After centrifugation, the supernatant was drained off, and the pellet was resuspended in a volume of serum-free DMEM, without antibiotics and glutamine, and incubated overnight at 4°C. The G418 and cell-culture reagents were purchased from Invitrogen (Burlington, ON, Canada), and the protamine sulfate and polybrene were purchased from Sigma (St Louis, MO, USA).

The MSC cultures were established from the bone marrow of 8-week-old KO mice, as previously described [26]. The MSCs were characterized using flow cytometry and antibodies specific for CD29 and CD44 (for the MSCs) and CD11b and CD45 (for the hematopoietic cells) and by the ability of the cells to differentiate into adipocytes and osteoblasts, following previously described methods [27].

The MSCs were transduced at two different MOIs (multiplicity of infection), always using 5 × 10^4^ cells plated on 25 cm^2^ dishes. The transduction was conducted for 24 hours in the presence of 10 μg/mL protamine sulfate. The medium was then replaced, and the cells were cultivated for 7 more days at 37°C in 5% CO_2_; the resulting culture medium was used to determine the IDUA activity. The MSC cultures demonstrating IDUA activity were allowed to grow for an additional 3 days in 75 cm^2^ dishes and used for the *in vivo* experiments. Cells at passage 3 and 4 were used for the *in vivo* experiments.

For transduction of the NIH3T3 mouse fibroblast cell line, the MSC transduction procedure was used. After transduction, the cells were selected with G418 (1 mg/mL) for at least 15 days. To determine the transduction efficiency of MSC with the *gfp* reporter gene, GFP-positive cells were counted using a fluorescence microscope.

### Alpha-L-iduronidase activity

Cells were collected after trypsinization, and the pellet was resuspended in homogenization buffer (10 mM NaPO_4_ pH 5.8, 0.1 mM DTT and 0.1% Triton X-100). The IDUA dosage protocol is described in M. Camassola *et al*. (submitted for publication). Briefly, in a 96-well microplate, 10 μL of each sample was mixed with 10 μL of 2.85 mM 4-methylumbelliferyl-alpha-L-iduronide (4MU-I, Toronto Research Chemicals, North York, ON, Canada) and 40 μL of 0.2 M formate buffer, pH 2.8. The plate was incubated at 37°C for 1 h, and the reaction was stopped by the addition of 175 μL of stop buffer (0.5 M glycine-NaOH, pH 10.3). The fluorescence of the 4MU product was determined using a Spectramax M2 fluorimeter (Molecular Devices, Sunnyvale, CA, USA) using 365 and 455 nm wavelengths for excitation and emission, respectively. The protein concentration was determined using the Bio-Rad protein dosage kit (Bio-Rad Laboratories, Hercules, CA, USA). The IDUA activity is expressed in units (U) representing nmols/h/mg of protein.

### GAG measurement

One third of the brain tissue was cut, ground in 10 volumes of acetone and left to stand overnight at room temperature. The acetone was changed every day over a period of five days and then discarded, and the sample was dried completely at 50°C. The dry weight was recorded, and the sample was incubated with 4 mg/mL maxatase in 0.05 M Tris–HCl buffer, pH 8.0, containing 0.15 M NaCl for 24 h at 60°C. Trichloroacetic acid was added (10% of final volume), and the sample was allowed to stand for 20 minutes at 4°C. The resulting precipitate was removed by centrifugation at 5000 × *g* for 20 minutes at room temperature. The GAGs from the supernatant were precipitated with two volumes of ethanol. After overnight incubation at −20°C, the GAGs were collected via centrifugation, vacuum dried and treated with deoxyribonuclease I (Sigma). The isolated GAGs were analyzed via agarose gel electrophoresis, using purified chondroitin sulfate (CS) and dermatan sulfate-heparan sulfate (DS-HS) as standards [28]. The quantification of the isolated GAGs was conducted using densitometry and Quick Scan 2000 Software (Beaumont, TX, USA). The results are expressed as μg/g of dry tissue or as the percentage of individual GAGs.

### Injection of transduced MSCs into the brain

This protocol was based on the study published by Coulson-Thomas and collaborators [29]. IDUA KO mice were anesthetized, and 1 × 10^6^ MLV-MSCs in 5 μL PBS were injected directly and slowly into the left ventricle of the brain using a 30-gauge needle. Five minutes after injection, the needle was removed, and the incision was sutured. Mice were left on a heating pad to recover and then transferred into micro-isolators. At the end of the experiments, the mice were euthanized, and the brains were removed and divided in 3 parts. One part was used for genomic DNA extraction, the second part was used for GAG determination and the last part was immediately processed with a Potter device (Eberbach Corporation, mod 7265.25, Ann Arbor, MI, USA) in 1 mL of homogenization buffer to determine the IDUA activity.

### Behavioral tests

All behavioral tests were conducted in the same room with controlled temperature and luminosity. The mice were separated into three groups, wild type (WT), knockout (KO) and knockout mice treated with IDUA KO MSCs transduced with MLV-IDUA (KO/IDUA). Before and after gene therapy, the animals underwent behavioral tests that focused on motor function and anxiety. The elevated plus maze test was conducted as previously described [16]. The mice were placed on a plus maze that had two walled arms (30 × 5 × 15 cm) and two open arms (30 × 5 cm) for 5 min. The spent time freezing and grooming and the number of times rearing and crossing (internal, external and total) were measured.

The maze was elevated 50 cm from the floor, and the animals were placed at the center to explore freely. The number of entries was counted, and the time spent in the closed or open arms was measured.

The open field test was performed based upon previous work [30] and is an observation of animals exposed only once to a circular arena with a diameter of 50 cm surrounded by a wall that is 40 cm high. The floor of the arena was divided into 19 equal squares, and the mice were placed at the center to explore freely. The time spent freezing and grooming and the number of times rearing and crossing (internal, external and total) were measured. Nonparametric Mann–Whitney *U* tests were used to compare the two variables. *p* values less than 0.05 were considered statistically significant.

### Molecular analyses

Genomic DNA (gDNA) was extracted from the brain using the QIAamp DNA Extraction Kit (QIAGEN, Hilden, Germany). PCR reactions for *IDUA* and an endogenous control were performed as previously described [30,31]. The PCR conditions used for *IDUA* detection were also used to detect *GFP* using the primers 5′-gag cct ggg gac ttt cca cac cc-3′ and 5′-gaa aat tgt gat gct att gc-3′.

### Immunohistochemistry

The animals were deeply anesthetized via an intraperitoneal injection of a ketamine and xylazine and intracardially perfused first with 0.9% saline solution and then with 4% paraformaldehyde in 0.1 M PBS. The brains were removed from the skull, fixed overnight at room temperature with 4% paraformaldehyde in 0.1 M PBS and cryoprotected with 0.1 M PBS containing 30% sucrose. The brains were frozen in isopentane (Sigma) using dry ice and embedded in Tissue Tek (Electron Microscopy Sciences, Hatfield, PA, USA). Midsagittal sections were cut with a cryostat (20 μm thickness), mounted on siliconized glass slides (DakoCytomation, São Paulo, Brazil) and fixed with 4% paraformaldehyde for 1 h. After thoroughly washing with PBS, the sections were first incubated with 0.1% Triton X-100 in PBS for 1 h at room temperature and then in blocking solution (10% BSA and 0.1% Triton X-100 in PBS) for 1 h at room temperature. The sections were then incubated with a rabbit anti-GFP antibody (1:500, Molecular Probes/Invitrogen, Eugene, OR, USA) overnight at 4°C. The next day, the sections were washed three times for 5 min each with PBS, incubated with DAPI (Molecular Probes/Invitrogen) and then incubated with an AlexaFluor 488–conjugated goat anti-rabbit antibody (1:200) in PBS for 1 h at room temperature. After washing three times in PBS for 5 min each, the sections were mounted with Fluormont-G (Electron Microscopy Sciences) and analyzed with fluorescence microscopy (Olympus BX60, Tokyo, Japan).

## Results

### Characterization of the IDUA KO MSCs and the transduction efficiency of the MLV vectors

**Figure 1:**
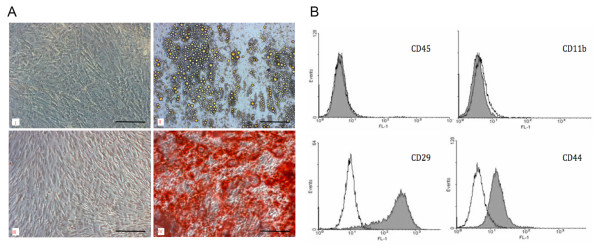
**Characterization of MSC cultures derived from IDUA KO mice.** When cultured in differentiation-inducing media, the MSCs readily differentiated into adipocytes (**A-II**) or osteoblasts (**A-IV**), as determined via staining with Oil Red O and Alizarin Red, respectively. Control cultures stained with the same reagents (Oil Red O or Alizarin Red in **A-I** and **A-III**, respectively) demonstrate the adherent, fibroblastoid morphology that characterizes the MSC cultures. The original magnification is 100×. As determined via immunophenotyping, the MSC cultures were negative for CD11b and CD45 and positive for CD29 and CD44. The MSCs and control cells are shown in grey and white, respectively (**B**).

The morphology of the MSCs isolated from the IDUA KO mice was characteristic of murine MSCs (Figure 1A). When exposed to inducing media, the MSCs differentiated into adipocytes and osteoblasts, as determined based on staining with Oil Red O and Alizarin Red, respectively (Figure 1A). Flow cytometric analyses demonstrated that the cells expressed surface markers characteristic of MSCs, such as CD29 and CD44, and lacked the expression of hematopoietic markers, such as CD11b and CD45 (Figure 1B).

To evaluate the transduction efficiency of the concentrated viral vectors, NIH3T3 cells were transduced at an MOI of 5.2 and selected with G418, after which the IDUA activity was evaluated (Table 1). These cells were used as a control for IDUA activity because the MLV-derived vectors have a high tropism for the NIH3T3 cell line, and these cells can be selected with G418 to obtain 100% IDUA-positive cells. Using these selected cells, we estimated the maximum level of IDUA gene expression and evaluated IDUA gene expression levels in the transduced MSCs.

**Table 1:** Transduction efficiency and gene expression analysis of MPSI-MSC and NIH3T3 cells with MLV-IDUA (I) and MLV-GFP (G) vectors

	MPSI-MSC^a^				NIH3T3^b^	
	MOI 5.2		MOI 77		G418	
	IDUA (U) ^c^	GFP (%)^d^	IDUA (U)^c^	GFP (%)^d^	IDUA (U)^c^	GFP (%)^d^
Control	ND	NE	ND	NE	23.8 ± 5.8	0
MLV-GFP	NE	NE	20.7 ± 21.6	NE	29.5 ± 29.5	100
1w (I/G)	2278 ± 249	8.9 ± 1.3	5340.4 ± 137.0	21.3 ± 6.6	NE	NE
3-4w (I/G)	3016.5 ± 780	9.3 ± 2.2	59.7 ± 16.8	ND	5849.1 ± 851.6	100
6w (I/G)	15 ± 1.4	ND	NE	ND	NE	NE

^a^ MPSI-MSC were transduced with 5.2 or 77 MOI of MLV vectors and evaluated for transgene expression during 6 weeks; ^b^ NIH3T3 cells were transduced with MLV vectors and selected with G418 (these cells were used as a positive control cells); ND = non detectable; NE = non-evaluated; w = week; ^c^ IDUA activity is expressed in units of activity (nmols/h/mg of protein); ^d^ GFP-positive cells are expressed in percentage. All experiments were performed at least in duplicates. (I/G) means to monitor expression of IDUA or GFP, MSC were transduced with MLV-IDUA or MLV-GFP, respectively.

The endogenous IDUA activity in non-transduced fibroblasts was at least 245 times lower than the activity in G418-selected cells (23.8 ± 5.8 U versus 5,849.1 ± 851.6 U; Table 1). The IDUA KO MSCs were transduced at an MOI of 5.2 or 77 to compare the transduction rate. One week after transduction, the cells transduced at an MOI of 77 presented a similar level of IDUA activity when compared to the transduced and selected NIH3T3 cells. However, after 4 weeks, production of IDUA by these cells decreased to basal levels. By contrast, IDUA KO MSCs transduced at an MOI of 5.2 had only 40% activity in the first week, and after four weeks, IDUA production increased to 51%, reaching 3016.5 U. However, 6 weeks after transduction, these cells also stopped producing IDUA, achieving just 15 U. The MSCs transduced with the MLV-GFP vector, which expressed only GFP and was used to determine the transduction rates, appeared to produce some level of IDUA (Table 1). However, this basal and non-specific activity could be due to the interference of GFP fluorescence, which emits a weak light signal at 455 nm.

The transduction rates, as determined by GFP expression, were similar to those of cells transduced with the MLV-IDUA vector (Table 1). G418-selected NIH3T3-GFP cells were 100% positive for GFP; whereas, one week after transduction, the IDUA KO MSCs transduced at an MOI of 5.2 or 77 MOI were only 8.9% and 21.3% GFP positive, respectively. After four weeks, the cells transduced at an MOI of 77 had no remaining GFP-positive cells, whereas the cells transduced at an MOI of 5.2 maintained the same percentage of GFP-positive cells that was detected 1 week after transduction. Six weeks after transduction, however, there were no detectable GFP-positive cells in the MSC cultures transduced at an MOI of 5.2, similar to the observed lack of IDUA activity in the cultures transduced with MLV-IDUA.

**Figure 2:**
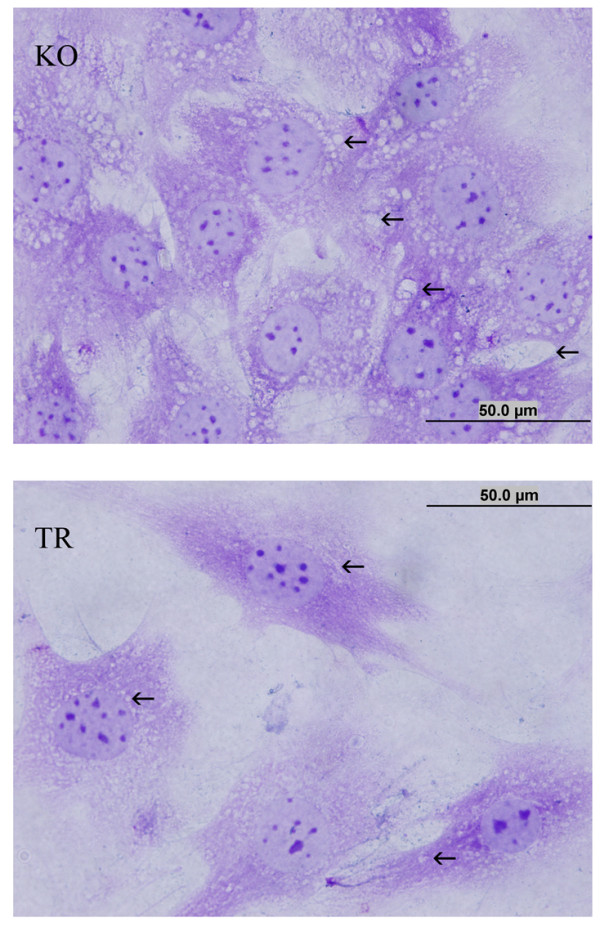
**Characterization of the vacuole size of MSCs transduced with MLV-IDUA by toluidine blue staining.** The arrows indicate the representative vacuoles for each group. The MSCs from the KO mice (KO) show many large vacuoles, while the MSCs transduced with MLV-IDUA (TR) show small vacuoles.

The MLV-IDUA-transduced MSCs were stained with toluidine blue to analyze vacuoles. The vacuole size of the treated cells was smaller than that of the non-treated cells, demonstrating some recovery of lysosomal function following the expression of the IDUA transgene (Figure 2).

### Behavioral evaluation

The WT and KO mice were tested in the open field and elevated plus maze tests before (Table 2) and after gene therapy (Table 3) to compare the exploratory behavior among the groups. In the open field test, the total number of events counted (total square) in younger WT mice (215.6) was much higher than the number in older WT mice (123), indicating hyperactivity in the younger mice. A similar profile of events was also observed in younger (157.2) and older (104.7) IDUA KO mice, but with lower overall frequencies than the number observed in the WT mice (Table 2). These results allow for the clear differentiation of young and old mice, as well as WT and IDUA KO mice. There were no significant variations in the freezing, grooming or rearing activities at these time points.

**Table 2:** Open field and elevated plus maze tests performed before applying gene therapy

		Open Field						Elevated plus maze			
		Freezing (s)	Grooming (s)	Rearing (#)	IS (#)	ES (#)	TS (#)	OA (#)	OA (s)	CA (#)	CA (s)
12 week-old mice											
WT (*n* = 5)	mean	9.4	10.4	46.4	79	136.6	215.6	2.8	29	9.2	223.2
	SD	5	6.7	10.8	22	25.3	44.8	1.5	24.9	3.3	23.4
KO (*n* = 10)	mean	15.1	12.1	32.2	42.5	114.7	157.2	2.8	42.9	6.4	194.5
	SD	9.6	6.7	14.1	14.3	38.5	42.6	2.8	47.4	4.3	60.2
					*p* <0.05		*p* < 0.05				
25 week-old mice											
WT (*n* = 3)	mean	25.3	10	37.3	49	74	123	2.3	55.3	8.3	182
	SD	15.9	4	24.1	34.7	15.9	50.4	0.6	64.7	5.7	113.6
KO (*n* = 5)	mean	24.4	9.1	23.7	33.9	70.9	104.7	2.6	25.9	4.5	193
	SD	10	6	13	13.6	38	46.4	3.1	25.9	3.0	49.4

**Table 3:** Open field and elevated plus maze tests performed after applying gene therapy

		Open Field							Elevated plus maze		
		Freezing (s)	Grooming (s)	Rearing (#)	IS (#)	ES (#)	TS (#)	OA (#)	OA (s)	CA (#)	CA (s)
20 week-old mice											
WT (*n* = 4)	mean	8.8	8	12.3	28.5	86.5	115	1.5	38.3	4.3	170.3
	SD	7.8	7.3	5.8	8.7	23.5	28.6	1.9	72.6	2.1	135.7
KO (*n* = 3)	mean	5,0	6,7	14,3	10,7	39,0	49,7	0,7	3,3	3,0	231,7
	SD	4,0	7,2	11,6	10,0	7,5	16,1	0,6	3,1	1,0	40,8
KO/IDUA (5)	mean	8.6	9.2	16.2	29.4	77.8	107.2	1	11.2	5.2	270.8
	SD	9.6	2.8	6.5	4	29	31.8	0.7	8.8	4.5	19.9
29 week-old mice											
WT (*n* = 3)	mean	8.7	9.3	6.7	17	45.3	62.3	0.3	1.7	2.7	286
	SD	5.5	8.1	3.8	5.3	27.6	32.9	0.6	2.9	1.5	3.5
KO (*n* = 4)	mean	5.0	5.2	1.7	8.7	23.5	32.2	1.2	93.5	1.5	93.5
	SD	2.9	4.1	2.4	7.6	11.4	18.0	1.3	130.2	1.3	124.5
KO/IDUA (3)	mean	5.7	5.7	11	16	56	72	0.7	4	3.3	283
	SD	2.1	2.1	14.2	13.1	45.2	58.3	1.2	6.9	2.1	14.2

Upon aging (to 29 weeks), the WT mice became less active (TS = 62.3), and the KO mice became even less active (TS = 32.2); however, the treated mice reached activity values similar to those for WT mice (TS = 72). The rearing activity of 29-week-old IDUA KO mice was decreased (1.7) when compared with WT mice (6.7) but was significantly improved after treatment (11). These results demonstrate a significant improvement in locomotive activity following gene therapy (Table 3).

In the elevated plus maze test, the WT mice showed the natural tendency of rodents to spend more time in the closed arm than the open arm. With older mice, this behavior is more evident; older mice spent 182 s in the closed arm and 223.2 s with younger mice. A similar profile was also observed with the number of entries into the arms (Table 2). These results were validated by the results of the opened-arm tendencies, which are approximately the inverse of those indicated by the closed-arm data. The KO mice displayed a behavior similar to that of young WT mice, spending more time in the closed arm (≈ 193 s) irrespective of age; however, at 29 weeks of age, the KO mice appeared very sick and spent most of their time in the closed arm (CA = 93.5 s). Surprisingly, the KO mice treated with gene therapy had a mobility similar to that of WT mice (CA = 286 s), even at 29 weeks (CA = 283 s) (Table 3).

### GAG Deposition

**Figure 3:**
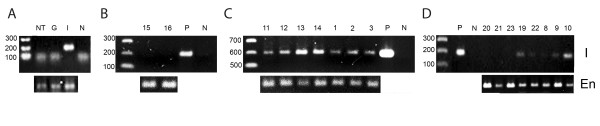
PCR analysis of transduced MSCs and treated mice. Genomic DNA isolated from MSCs from KO mice (**A**), brains from untreated KO mice (**B**), brains from wild-type mice (**C**), and brains from KO mice treated with MLV-IDUA-transduced MSCs (**D**) was used to analyze the *IDUA* and *β-actin* genes. The three molecular markers in the figures represent 300, 200 and 100 bp. N = PCR negative control; P = PCR positive control; I = IDUA; En = Endogenous β-actin; NT = non-transduced cells. The numbers on the figures indicate the number of each animal.

At the end of the behavioral evaluations, the animals were euthanized, and the brains were removed for cellular and molecular analyses. IDUA activity was undetectable in any of the animals. PCR amplification of the *IDUA* sequence from genomic DNA showed the presence of the expected DNA fragment in almost all treated mice, except for four samples (Figure 3).

**Figure 4:**
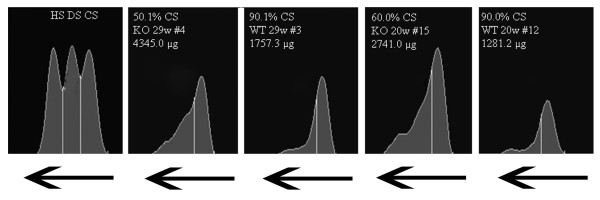
**Electrophoretic analysis of GAGs extracted from the brains of 20- and 29-week-old IDUA KO and WT mice.** The GAGs extracted from mouse brains were separated via electrophoresis to identify chondroitin (CS) and dermatan (DS)-heparan (HS) sulfate, and the band of each GAG was quantified by densitometry. The total GAG amount is expressed as μg/g of dry tissue. The phenotype, age group, animal identification number (#) and percentage of CS are indicated in the figure. The arrows indicate the direction of movement of the GAG sample during gel electrophoresis.

Increased GAG deposition was visible in the KO mice when compared with the WT mice, and GAG deposition increased with age (Figure 4 and Table 4): from 1566 in WT at 20 w and 1907 in WT at 29 w to 2443 in KO at 20 w and 3234 in KO at 29 w. The treatment with IDUA-expressing MSCs in young mice resulted in decreased GAG deposition (2065) to a level that was similar to the level observed in WT mice at 29 w. In treated older KO mice, the decrease was more significant (1509), reaching a level similar to the level in the younger WT mice at 20 w. Despite the dramatic reduction in total GAG deposition following gene therapy, the proportion of dermatan and heparan sulfate in the total GAG content in the KO mice did not change significantly after treatment and remained higher than level observed in the WT mice.

**Table 4:** GAG concentration in brains of mice treated with gene therapy

#	Groups	Total GAG	Mean ± SD	DS-HS (%)^a^
11	WT 20w	1140.4	1566 ± 419	6.2
12	WT 20w	1281.2		9.9
13	WT 20w	1997.3		32.1
14	WT 20w	1849.6		29.0
1	WT 29w	NE	1907 ± 212	23.8
2	WT 29w	2057.0		NE
3	WT 29w	1757.3		9.8
15	KO 20w	2741.0	2443 ± 470	40.0
16	KO 20w	2889.3		48.8
24	KO 20w	1850.0		50.0
25	KO 20w	2290.0		48.2
4	KO 29w	4345.0	3234 ± 1287	49.8
5	KO 29w	3235.5		47.7
26	KO 29w	1429.0		43.5
27	KO 29w	3930.0		57.3
19	KO/IDUA 20w	1220.2	2065 ± 487	37.3
20	KO/IDUA 20w	2323.0		42.1
21	KO/IDUA 20w	2404.4		46.1
22	KO/IDUA 20w	2296.0		49.2
23	KO/IDUA 20w	2083.0		49.4
8	KO/IDUA 29w	1656.0	1509 ± 364	42.6
9	KO/IDUA 29w	1778.9		39.8
10	KO/IDUA 29w	1094.2		55.6

Total GAG is expressed in μg/g dry tissue; (#) Animal identification number;

^a^ % = [DS-HS]/[Total GAG] * 100; NE = non-evaluated; w = week-old.

### MSC migration

**Figure 5:**
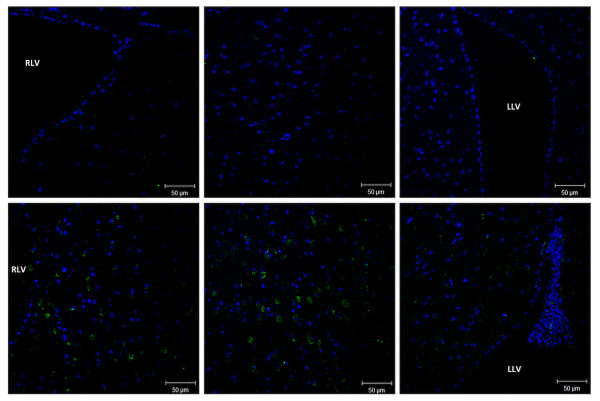
Immunohistochemistry of mouse brains injected with GFP-expressing MSCs. The MSCs transduced with MLV-GFP were injected into mouse brains and analyzed via IHC 1 week later. Images of the right ventricle (RLV), the left ventricle (LLV) and the middle ventricular area are shown.

To evaluate the migration of the injected MSCs in the brain, cells transduced with the MLV vector carrying GFP were injected into the brain following the same procedure. The presence of MSCs in the brain ventricles is clearly detected after one week. However, even though the cells were injected into the left ventricle, GFP-positive cells were detected in all of the ventricles (Figure 5), demonstrating the mobility of these cells in the ventricles.

## Discussion

In gene therapy studies, the characterization of the level and time of gene expression for each vector is the first and fundamental step for the validation of the studies because these factors depend on the promoter/enhancer used for vector construction, the vector formulation, the therapeutic gene, the targeted cells and the administration route. Previous work has demonstrated that human MSCs are poorly transduced with MLV vectors [32]; consequently, a high MOI is used to increase the transduction efficiency, with the expectation of a high-level gene expression. To estimate the MOI necessary to obtain a high level of *IDUA* gene expression, MSCs were infected at two MOI and compared with NIH3T3 cells transduced with the same vector and selected with G418 to obtain 100% genetically modified cells. An MOI of 77 resulted in a transduction efficiency of 21%. However, curiously, this percentage was sufficient to reach the level of IDUA produced by the selected NIH3T3 cells (Table 1), suggesting a higher production of IDUA per cell in the MSCs when compared with NIH3T3 cells. Additionally, an MOI of 5.2 resulted in a transduction efficiency of 10%, and the transduced MSCs produced approximately 50% of the IDUA when compared with the cells transduced at an MOI of 77. These results indicate that the concentration of virus required to increase the transduction efficiency is not linear and that the level of *IDUA* gene expression per cell is highly variable among different cell types.

As we aimed to use long-term gene expression to correct a permanent IDUA deficiency in our study, the MLV-transduced MSCs were monitored for more than a month by measuring IDUA activity as well as by counting the number of GFP-positive cells. The IDUA activity in MSCs transduced at an MOI of 77 decreased to the basal level in 4 weeks, and this result was confirmed by the absence of GFP-positive cells at this time. However, the MSCs transduced at an MOI of 5.2 maintained transgene expression two weeks longer than the MSCs transduced at the higher MOI, as verified both by the IDUA activity and the presence of GFP-positive cells. Silencing of gene expression after MLV transduction is a phenomenon that is known, especially in progenitor cells [33-35]. Our results indicate that this silencing is accentuated at higher MOI. Even though the MSCs transduced at the higher MOI produced twice the amount of IDUA when compared with the cells transduced at the lower MOI, we decided to use the lower MOI in the remaining experiments for biosafety reasons [36,37] and for better long-term gene expression.

The main goal of our gene therapy study was to reduce GAG deposition in the brain after the ventricular injection of MSCs transduced with MLV-IDUA; we expected an improvement in exploration as a consequence of the gene therapy. The injection of the MSCs was not difficult and was reproducible, even in mice weighing 20–25 g, and the mortality caused by the injection procedure was low. A single injection of MSCs in the left ventricle was enough to distribute these cells evenly in all ventricular areas (Figure 5), which is a very important experimental finding that can be used for translation to a clinical assay in the future.

In our study, we used adult mice because MPSI pathology is already established in adults, and therefore, these mice mimic the situations observed in human patients. To validate our study, the behavioral characteristics were initially analyzed. As neurodegeneration is a progressive and slow process that affects animal behavior, there is no specific age at which the animal can be diagnosed as fully affected. Therefore, it is very important to determine the behavioral state of KO animals before initiating gene therapy experiments. To evaluate the evolution of MPSI disease and aging, two behavioral tests were used in our study, the open field and elevated plus maze tests, which monitor exploratory activity and anxiety, respectively. In this study, the hyperactivity of young WT mice was clearly observed in the open field test, with measurements of 215.6 events in total squares that decreased to 123 events with age (Table 2). In KO mice, reduced movement was evident when compared with WT mice, and the movement decreased further with age. A similar reduction was observed in rearing behavior in both the old and KO mice. These results indicate that exploratory activity, which requires good neuromuscular function, is dependent on age and MPSI disease progression. However, the anxiety and cognitive levels, as determined via the elevated plus maze test, seemed to be less affected in younger KO mice (CA = 231.7 s) but strongly affected in older KO mice (CA = 93.5 s) (Table 3). These findings suggest that MPSI somehow initially affects neuromotor activity more than the activity of the central nervous system. These results also demonstrate that sick IDUA KO mice can be easily identified via the open field and the elevated plus maze tests, which should be considered important tests for monitoring the evolution of this disease and the efficacy of gene therapy treatments.

*Ex vivo* transfer of *IDUA* via the injection of transduced MSCs directly into the brain resulted in a clear improvement in the behavioral tests: the treated mice behaved like age-matched WT in both the open field and elevated plus maze tests (Table 3). The older treated mice were 29 weeks old at the time of the test, and at this age, most sick mice become very weak; some die because of the advanced illness. Therefore, the behavioral improvements detected in our study are highly relevant because the treated mice behaved like WT mice, even at an advanced age.

To understand the observed behavioral improvements, GAG deposition in the brain was analyzed. One possible mechanism underlying the neuropathological progression in these animals, and in IDUA patients, is the progressive accumulation of GAGs, which occurs due to the lack of IDUA activity [5]. The IDUA KO mice normally present with approximately 60% more GAGs when compared with WT mice (1566 μg to 2443 μg for 20-week-old mice and 1907 μg to 3232 μg for 29-week-old mice); however, the treated young mice displayed an intermediate level of GAGs, which can explain the improved behavioral performance. In the older treated KO mice, the reduction in GAG deposition was more drastic, reaching the lowest observed level (1509 μg). Once again, the reduced GAG deposition likely underlies the improved behavioral performance (Table 4). Collectively, these data demonstrate a strong relationship between GAG accumulation in the brain and behavioral changes, which can be improved by the administration of MSCs expressing IDUA directly into the brain. Importantly, this improvement can be seen even in advanced disease.

The reduction in GAG deposition suggests the presence of IDUA activity; however, enzymatic activity was not detected in the brains of the treated mice. This is somewhat expected as in the *in vitro* study, IDUA activity was detected up to 4 weeks after transduction of MSCs with MLV-IDUA, and no activity was observed 6 weeks after transduction (Table 1). As the injected MSCs are in a different environment, transgene expression can be shut down early, and the MSC survival time could be shorter. Therefore, the reduction in GAG deposition observed 1 or 2 months after injection of IDUA-expressing MSCs is likely the consequence of high IDUA production at the beginning of the treatment, which has an unknown duration.

As was previously discussed, ERP and bone marrow transplantation do not slow neurodegeneration in MPSI patients. Consequently, in our opinion, direct treatment of the brain is necessary. With the present study, we demonstrate that IDUA-expressing MSCs are a good therapeutic candidate to treat neurodegeneration via direct injection into the brain ventricles. This procedure can be performed in MPSI patients by applying these cells via the cannula used to drain liquid from the brain of these patients.

## Competing interests

The authors declare that they have no competing interests.

## Authors' contributions

FHS, MC constructed vectors; FHS, MC, NBN, PKM, RSS prepared and characterized MSC cells; FHS, VGP, EGY, LZT, LPC, BCGL, PKM, RSS, VYS, BFAC, LCB, VA, TRMF, MP participated in animal experiments; FHS, LT, HBN carried out molecular analyses and VYS, FHS histology; SWH, FHS designed and drafted manuscript and SWH supervised all studies. All authors read and approved the final manuscript.
